# Dihydroquinine Enhances Radiosensitivity in Cervical Cancer Cells Accompanied by Radiation-Induced Cellular Responses

**DOI:** 10.3390/cells15161484

**Published:** 2026-08-18

**Authors:** Ausanai Prapan, Pimvaree Aissara, Peerawit Soonthornchookiat, Chanyatip Suwannasing, Jirapas Jongjitwimol, Chatrawut Pattaweerakul, Yu Xiong, Saranya Chaiwaree, Hans Bäumler

**Affiliations:** 1 Department of Radiological Technology, Faculty of Allied Health Sciences, Naresuan University, Mueang District, Phitsanulok 65000, Thailand; ausanaip@nu.ac.th (A.P.); pimv2544@gmail.com (P.A.); peerawits@nu.ac.th (P.S.); sawitreesu@nu.ac.th (C.S.); 2 Department of Medical Technology, Faculty of Allied Health Sciences, Naresuan University, Mueang District, Phitsanulok 65000, Thailand; jirapasj@nu.ac.th; 3 Department of Radiology, Faculty of Medicine, Naresuan University, Mueang District, Phitsanulok 65000, Thailand; chatrawoot@gmail.com; 4 Institute of Transfusion Medicine, Charité-Universitätsmedizin Berlin, 10117 Berlin, Germany; yu.xiong@charite.de; 5 Department of Pharmaceutical Technology and Biotechnology, Faculty of Pharmacy, Payap University, Chiang Mai 50210, Thailand; saranya_c@payap.ac.th

**Keywords:** dihydroquinine, radiosensitization, cervical cancer, reactive oxygen species, radiotherapy

## Abstract

**Highlights:**

**What are the main findings?**

- Dihydroquinine (DHQ) enhanced radiosensitivity and reduced clonogenic survival in irradiated cervical cancer cells.
- DHQ increased radiation-induced cellular responses by increasing ROS, DNA damage signaling, and apoptosis.

**What are the implications of the main findings?**

- DHQ may represent a promising candidate for radiosensitizer development.
- These findings provide a basis for further mechanistic and preclinical investigation of DHQ as a potential radiosensitizer.

**Abstract:**

Radiosensitizers are being investigated to improve the therapeutic efficacy of radiotherapy by enhancing tumor cell sensitivity while minimizing damage to normal tissues. Dihydroquinine (DHQ), a naturally occurring Cinchona alkaloid with diverse biological activities, has not previously been evaluated for radiosensitizing potential. In this study, human cervical cancer (HeLa) cells were pretreated with an IC20 concentration of DHQ and exposed to 6 MV X-ray irradiation. Clonogenic survival was assessed after irradiation at 2, 4, and 6 Gy, while intracellular ROS, γ-H2AX immunofluorescence, and apoptosis were evaluated following DHQ pretreatment and 2 Gy irradiation. DHQ pretreatment reduced clonogenic survival, yielding sensitizer enhancement ratio values of 1.37 and 1.55 at surviving fractions of 0.20 and 0.37, respectively, indicating modest-to-moderate enhancement of radiosensitivity. DHQ was also associated with increased ROS production, elevated γ-H2AX positivity, and enhanced apoptosis compared with irradiation alone. These findings suggest that DHQ enhances the radiation response in HeLa cells and is associated with increased radiation-induced cellular responses. Although the radiosensitizing effect was modest, the consistent findings across multiple biological endpoints support further investigation of DHQ as a potential adjunct to radiotherapy. Further studies are warranted to validate these findings in more clinically relevant preclinical models.

## 1. Introduction

Cervical cancer remains a major global health issue and is one of the leading causes of cancer-related illness and death among women worldwide [1,2]. Despite advances in screening programs and prophylactic human papillomavirus (HPV) vaccination, a substantial proportion of patients are still diagnosed at locally advanced stages, at which radiotherapy plays a central role in both definitive and adjuvant treatment [3]. However, the therapeutic efficacy of radiotherapy is often limited by intrinsic or acquired radioresistance, leading to treatment failure, local recurrence, and disease progression [4,5]. Therefore, improving tumor radiosensitivity without increasing normal tissue toxicity remains a critical challenge in cervical cancer management.

Radiosensitizers are agents that improve the biological effects of ionizing radiation, thereby improving tumor control at clinically acceptable radiation doses [6,7]. In cervical cancer, concurrent cisplatin-based chemoradiotherapy (CCRT) remains the current standard of care for patients with locally advanced cervical cancer [8,9]. Cisplatin enhances radiation response primarily through interference with DNA repair pathways and cell cycle regulation. However, its clinical application is often limited by significant toxicities, including nephrotoxicity, gastrointestinal complications, and hematological suppression [10]. These limitations have driven growing interest in developing alternative radiosensitizers with improved safety profiles and novel mechanisms of action.

At the cellular level, ionizing radiation mainly causes DNA damage, particularly DNA double-strand breaks (DSBs), which are the most lethal type of radiation damage. In addition to directly ionizing DNA, radiation generates reactive oxygen species (ROS) via water radiolysis, thereby causing indirect DNA damage and oxidative stress [11,12]. Tumor cells with higher antioxidant defenses or better DNA repair capacity are more likely to survive radiation exposure, thereby contributing to radioresistance [13]. Therefore, agents that elevate intracellular ROS levels or interfere with DNA repair pathways are promising strategies for enhancing radiosensitization.

Natural compounds are gaining more attention as possible radiosensitizers because of their varied biological effects and generally better toxicity profiles compared to traditional chemotherapeutic drugs [14]. Several phytochemicals, such as curcumin, resveratrol, and quercetin, enhance radiation-induced cytotoxicity by amplifying DNA damage, promoting apoptosis, and modulating oxidative stress through antioxidant responses, thereby influencing mitochondrial function and apoptotic signaling pathways in cancer radiotherapy [15]. These findings support continued investigation of naturally occurring compounds as potential adjuncts to radiotherapy.

Dihydroquinine (DHQ; also known as Hydroquinine) is a naturally occurring Cinchona alkaloid structurally related to quinine, a compound historically used as an antimalarial agent [16]. DHQ possesses a quinoline-based molecular framework, a characteristic feature of many biologically active alkaloids. Quinoline-containing compounds have been reported to modulate cellular redox homeostasis, mitochondrial function, and stress-response pathways, suggesting a potential role in influencing radiation-induced oxidative stress and radiosensitivity. Although quinine and its derivatives have been extensively investigated for their antiparasitic and pharmacological activities, the biological properties of DHQ remain comparatively underexplored, particularly in the context of cancer biology and radiation response.

Previous studies have demonstrated that Cinchona alkaloids can modulate intracellular redox balance, mitochondrial function, and apoptotic pathways [17], which are key determinants of cellular responses to ionizing radiation. In addition, DHQ has been reported to exhibit antimicrobial activity against multidrug-resistant microorganisms, indicating its potential to modulate biological responses under stress conditions [18,19]. Furthermore, transcriptomic and phenotypic analyses have shown that DHQ can modulate cellular regulatory pathways associated with motility, biofilm formation, and gene expression in microbial systems [20]. Collectively, these observations suggest that DHQ may influence cellular stress responses through multiple biological mechanisms, thereby supporting its potential significance in modulating radiation-induced cellular effects. Unlike extensively studied phytochemical radiosensitizers such as curcumin, resveratrol, and quercetin, the potential role of DHQ in radiation response remains underexplored. Given its quinoline-based alkaloid structure and reported effects on redox regulation, mitochondrial function, and apoptosis-related pathways, DHQ may influence radiation-induced oxidative stress and cellular stress responses. Therefore, DHQ represents an interesting but largely unexplored candidate for radiosensitization studies.

Given that ionizing radiation exerts its cytotoxic effects predominantly through the generation of ROS and DNA damage, agents that disrupt redox homeostasis or interfere with DNA repair mechanisms may augment tumor radiosensitivity. However, despite the reported biological activities of DHQ, its influence on cancer cell response to ionizing radiation has not been systematically investigated. This represents an important knowledge gap in current radiosensitizer research. To the best of our knowledge, this is the first study to evaluate the potential of DHQ as a radiosensitizer in cancer cells. Therefore, this study aimed to investigate the radiosensitizing effect of DHQ in human cervical cancer cells following clinically relevant X-ray irradiation. Clonogenic survival was selected as the primary endpoint, while intracellular ROS generation, γ-H2AX expression, and apoptosis were evaluated to characterize the biological responses associated with the combined DHQ and irradiation treatment.

## 2. Materials and Methods

### 2.1. Chemicals and Reagents

Dihydroquinine, also known as hydroquinine (DHQ; ≥98% purity), was purchased from Sigma-Aldrich (St. Louis, MO, USA). DHQ stock solutions were prepared in 30% (*v*/*v*) dimethyl sulfoxide (DMSO), with working solutions freshly diluted in complete culture medium just before each experiment. During the MTT assay, the DMSO concentration never exceeded 1.5% (*v*/*v*). For all subsequent experiments, DHQ was used at the IC20 concentration from the MTT assay, resulting in a final DMSO concentration not exceeding 0.75% (*v*/*v*). This concentration was selected to minimize potential solvent-related effects while maintaining adequate DHQ solubility.

### 2.2. Cell Culture and Treatment Groups

Human cervical cancer cells (HeLa; RIKEN BRC, RCB0007) were kindly provided by Prof. Felicity Z. Watts (University of Sussex, Brighton, UK). Human dermal fibroblasts (CCD-1123Sk; ATCC) were kindly provided by Assoc. Prof. Dr. Kanchana Usuwanthim (Naresuan University, Phitsanulok, Thailand). Both cell lines were grown in Dulbecco’s Modified Eagle Medium (DMEM) with 10% fetal bovine serum (FBS), 100 U/mL penicillin, and 100 µg/mL streptomycin. Cells were maintained at 37 °C in a humidified atmosphere with 5% CO_2_ and routinely subcultured at 70–80% confluence. For experimental treatments, HeLa cells were assigned to four groups: (1) untreated control cells, (2) cells treated with DHQ alone, (3) cells exposed to X-ray irradiation alone, and (4) cells pretreated with DHQ for 24 h followed by X-ray irradiation.

### 2.3. DHQ Treatment and Determination of IC20 Concentrations

To determine an appropriate concentration for radiosensitization experiments, the impact of DHQ on cellular metabolic activity was initially assessed through the MTT assay, which uses 3-(4,5-dimethylthiazol-2-yl)-2,5-diphenyltetrazolium bromide. HeLa cells were plated in 96-well plates at 1 × 10^4^ cells per well and exposed to 0–50 µg/mL DHQ for 24 h. Following treatment, cells were incubated with MTT reagent (0.5 mg/mL) for 3 h at 37 °C. The resulting formazan crystals were dissolved in DMSO, and absorbance was measured at 570 nm using a microplate reader. Cellular metabolic activity was expressed as a percentage relative to untreated control cells, and the IC20 was determined from dose–response curves. Based on these results, the IC20 concentration of DHQ was selected for subsequent radiosensitization experiments. Cells in the combined-treatment group were pretreated with DHQ for 24 h before X-ray irradiation. To provide preliminary information on the response of non-malignant cells to DHQ, human fibroblasts were treated under the same experimental conditions used for HeLa cells. Cells were exposed to the indicated concentrations of DHQ for 24 h, and cellular metabolic activity was determined using the MTT assay. Untreated cells and corresponding DMSO vehicle controls were included. These results are presented in Supplementary Figure S1.

### 2.4. Irradiation Setup

Irradiation was carried out with a 6 MV X-ray photon beam produced by a clinical linear accelerator (Varian Clinac 2100C; Varian Medical Systems, Palo Alto, CA, USA). HeLa cells were exposed to radiation doses of 2, 4, and 6 Gy for the clonogenic survival assay, while a single dose of 2 Gy was used for all other experiments. During irradiation, culture plates were positioned horizontally at the isocenter within a tissue-equivalent bolus phantom. An additional 1.5 cm bolus was placed on top of the plates to ensure adequate dose buildup and electronic equilibrium. The cell monolayer was positioned at an effective depth of 3.5 cm. The source-to-surface distance (SSD) was maintained at 96.5 cm, and a 20 × 15 cm^2^ field size with a vertical photon beam was used to ensure uniform dose delivery across all samples.

### 2.5. Clonogenic Survival Assay and SER Calculation

The effect of DHQ in combination with ionizing radiation on the clonogenic survival of HeLa cells was evaluated using a colony formation assay [21]. Briefly, 2 ×10^5^ HeLa cells were seeded into 6-well plates and allowed to adhere overnight. Cells were then treated with DHQ at the IC20 concentration for 24 h, followed by exposure to single X-ray doses of 2, 4, and 6 Gy. After treatment, cells were harvested, counted, and seeded into 6-well plates at densities optimized based on expected cytotoxic effects: 150 cells/well for untreated control cells, 300 cells/well for irradiation-only treatment, and 300 cells/well for the combined DHQ and irradiation treatment. Cells were incubated in a humidified atmosphere at 37 °C with 5% CO_2_ for 10–14 days, and colony development was monitored during this period. The exact endpoint varied among the three independent experiments depending on colony development; however, within each independent experiment, all treatment groups were fixed, stained, and scored at the same time point. Cultures were not maintained beyond 14 days. Colonies were subsequently fixed with methanol for 45 min, stained with 0.5% crystal violet (Sigma-Aldrich, St. Louis, MO, USA) for 30 min, and rinsed gently with distilled water. Colonies containing more than 50 cells were counted. The plating efficiency (PE) was calculated as the ratio of the number of colonies formed to the number of cells seeded in the non-irradiated control group. The survival fraction (SF) was determined as the number of colonies counted divided by the product of the number of cells seeded and the PE. Survival curves were generated using the linear–quadratic (LQ) model [22] expressed by the following formula:

SF = exp(−(αD + βD^2^)) (1)

where SF is the surviving fraction, D is the radiation dose, and α and β are the linear and quadratic components of cell killing, respectively.

The sensitization enhancement ratio (SER) is conventionally calculated using the dose required to achieve a surviving fraction (SF) of 0.1 (D10), as this endpoint is widely used to evaluate radiosensitizing effects in radiobiological studies [23]. However, in the present study, the clonogenic survival curves did not reach SF = 0.1 within the investigated dose range. Therefore, to avoid unreliable extrapolation beyond the experimental data, SER was determined using the doses required to achieve surviving fractions of 0.20 (D20) and 0.37 (D37), obtained from the fitted survival curves. SER was calculated as follows:

(2) $$\mathrm{SERx}\;=\frac{{\mathrm{Dx}}_{\;\mathrm{radiation}\;\mathrm{only}}}{{\mathrm{Dx}}_{\;\mathrm{DHQ}+\mathrm{radiation}}}$$

where Dx represents the radiation dose required to achieve a surviving fraction of x. Accordingly, SER20 and SER37 were calculated at surviving fractions of 0.20 and 0.37, respectively.

### 2.6. Intracellular ROS Measurement

Intracellular reactive oxygen species (ROS) were assessed using the fluorescent probe 2′,7′-dichlorodihydrofluorescein diacetate (DCFH-DA). HeLa cells (1 × 10^5^ cells/well) were seeded in 24-well plates and incubated for 24 h to allow cell attachment. Cells were then pretreated with DHQ at an IC20 concentration for 24 h, followed by a single 2 Gy X-ray dose. After treatment, cells were incubated with DCFH-DA at a final concentration of 20 µM for 30 min at 37 °C in the dark. Cells were subsequently washed with PBS to remove excess dye, and fluorescence intensity was measured using a microplate reader with excitation and emission wavelengths of 485 nm and 535 nm, respectively. The fluorescence intensity of dichlorofluorescein (DCF) was used as an indicator of intracellular ROS levels. Results were expressed as mean fluorescence intensity relative to the untreated control group. All experiments were conducted in triplicate.

### 2.7. γ-H2AX Immunofluorescence Assay

HeLa cells (1 × 10^5^ cells/well) were seeded in 24-well plates and incubated for 24 h to promote attachment. Cells were pretreated with DHQ at the IC20 concentration for 24 h, followed by a single 2 Gy X-ray dose. One hour after irradiation, γ-H2AX immunofluorescence staining was used to assess DNA double-strand breaks. Briefly, cells were washed with PBS, fixed in 4% paraformaldehyde for 30 min, permeabilized with 0.5% Triton X-100 in PBS for 7 min, and blocked with 2% bovine serum albumin (BSA; Sigma-Aldrich; Merck KGaA, Darmstadt, Germany) in PBS for 1 h. They were then incubated overnight at 4 °C with a mouse monoclonal anti-phospho-histone H2AX (Ser139) primary antibody (Thermo Fisher Scientific, Waltham, MA, USA) at a 1:300 dilution. After three PBS washes, cells were treated with Alexa Fluor 488-conjugated goat anti-mouse IgG secondary antibody (Gibco, Thermo Fisher Scientific, Waltham, MA, USA) at a 1:400 dilution for 1 h at room temperature. Nuclei were counterstained with DAPI (Gibco), and images were captured using a Carl Zeiss fluorescence microscope (Zeiss, Jena, Germany). For each sample, three randomly selected microscopic fields were imaged, and two blinded investigators manually counted the total number of nuclei and γ-H2AX-positive cells. A cell was classified as γ-H2AX-positive when detectable γ-H2AX fluorescence overlapped with the DAPI-stained nuclear region in the merged image, whereas cells without detectable nuclear γ-H2AX fluorescence were classified as negative. Individual γ-H2AX foci were not counted. The percentage of γ-H2AX-positive cells was calculated for each field and averaged across the three fields for statistical analysis. Accordingly, γ-H2AX immunofluorescence was evaluated as the percentage of positive cells, providing a semi-quantitative assessment of DNA damage signaling.

### 2.8. Apoptosis Analysis

HeLa cells (1 × 10^5^ cells/well) were seeded in 24-well plates and incubated for 24 h to promote cell attachment. Then, cells were pretreated with DHQ at the IC20 concentration for 24 h, followed by a single 2 Gy X-ray dose. After irradiation, cells were incubated for another 24 h. Subsequently, cells were harvested, and the populations of viable, early apoptotic, late apoptotic, and dead cells were quantitatively analyzed using the Muse^®^ Cell Analyzer (EMD Millipore Corp., Burlington, MA, USA) with the Annexin V and Dead Cell Assay kit and software module (EMD Millipore Corp., Burlington, MA, USA), following the manufacturer’s instructions. All experiments were conducted in triplicate.

### 2.9. Statistical Analysis

All data are expressed as mean ± standard deviation (SD). Statistical analysis was performed using one-way or two-way ANOVA, followed by Tukey’s post hoc test as needed. All analyses and graphs were produced with GraphPad Prism software (version 11; GraphPad Software, Inc., San Diego, CA, USA). Results with a *p*-value of less than 0.05 were considered statistically significant.

## 3. Results

### 3.1. Determination of IC20 Concentration of DHQ

**Figure 1:**
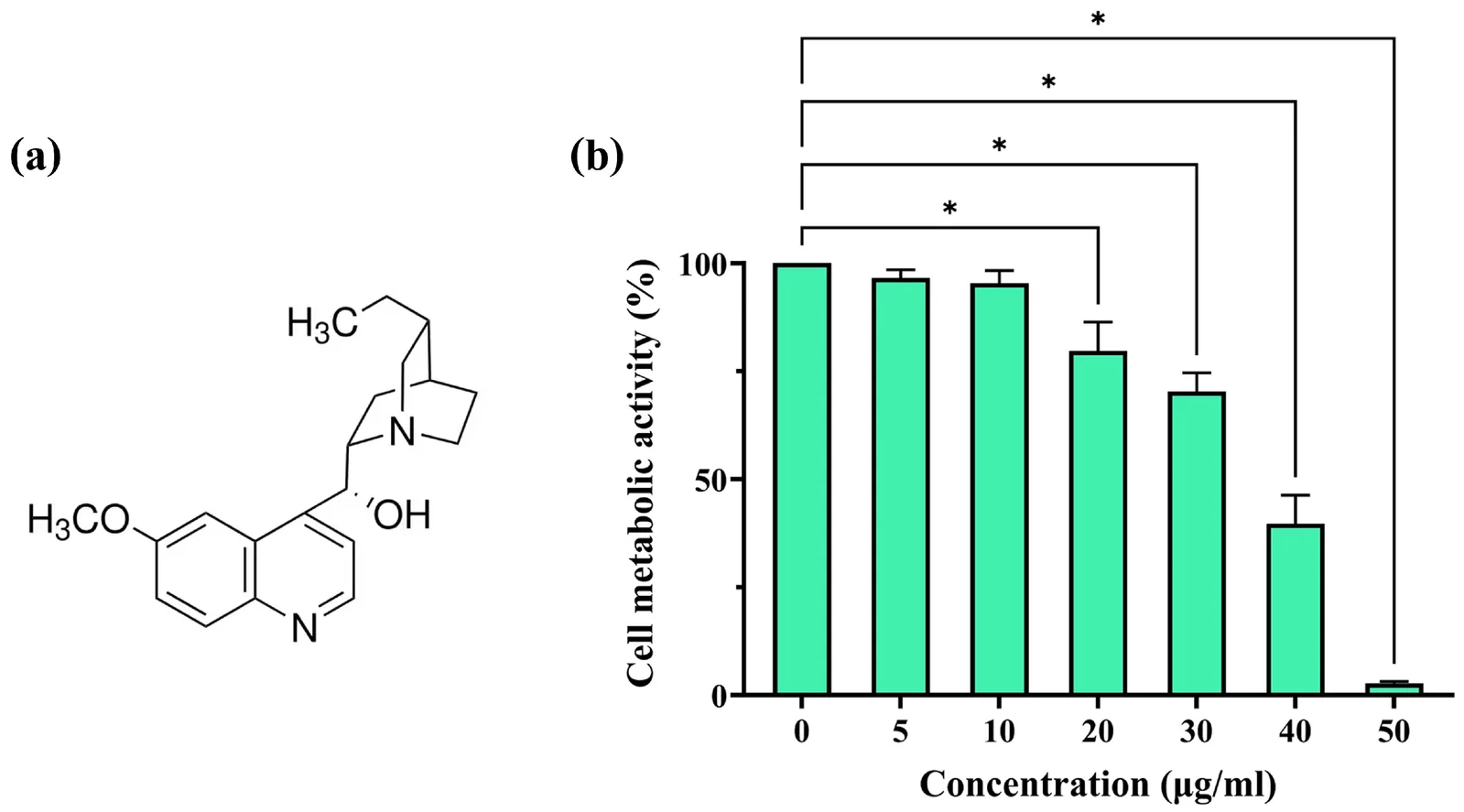
Chemical structure and cell viability of dihydroquinine (DHQ). (**a**) Chemical structure of DHQ, a quinoline-derived alkaloid obtained from Cinchona species (molecular formula: C_20_H_26_N_2_O_2_; molecular weight: 326.43 g/mol). (**b**) Cell metabolic activity of HeLa cells following exposure to DHQ (0–50 µg/mL) for 24 h, determined using the MTT assay. A concentration-dependent reduction in cell viability was observed, and the IC20 value (25.23 µg/mL) was selected for subsequent radiosensitization experiments. Data are presented as mean ± SD (*n* = 3). * *p* < 0.05.

The chemical structure of DHQ is shown in Figure 1a. The cytotoxic effects of DHQ on HeLa cells were assessed using the MTT assay after 24 h of exposure (Figure 1b). DHQ induced a clear dose-dependent reduction in cellular metabolic activity. At lower concentrations, viability remained high, with 96.6 ± 1.3% at 5 µg/mL, 94.5 ± 2.8% at 10 µg/mL, and 76.9 ± 4.3% at 20 µg/mL. Further reductions were observed at intermediate concentrations, with viability decreasing to 68.4 ± 1.9% at 30 µg/mL and 39.7 ± 6.6% at 40 µg/mL. At higher concentrations, DHQ exhibited marked cytotoxicity, reducing cell viability to 2.8 ± 0.4% at 50 µg/mL. Furthermore, nonlinear regression analysis of the dose–response curve indicated that the IC20 value of DHQ was 25.23 µg/mL, corresponding to approximately 80% cell viability. This concentration was therefore selected for subsequent experiments as an IC20 condition to minimize the direct cytotoxic effects of DHQ while enabling evaluation of its combined effect with ionizing radiation. To provide preliminary information on the response of non-malignant cells to DHQ under the same experimental conditions, cellular metabolic activity was evaluated in human fibroblasts (Supplementary Figure S1). At concentrations around the IC20 used in HeLa cells, fibroblast cellular metabolic activity remained above approximately 80% after 24 h of treatment.

### 3.2. DHQ Increases Radiation-Associated Clonogenic Cell Death and SER Calculation

**Figure 2:**
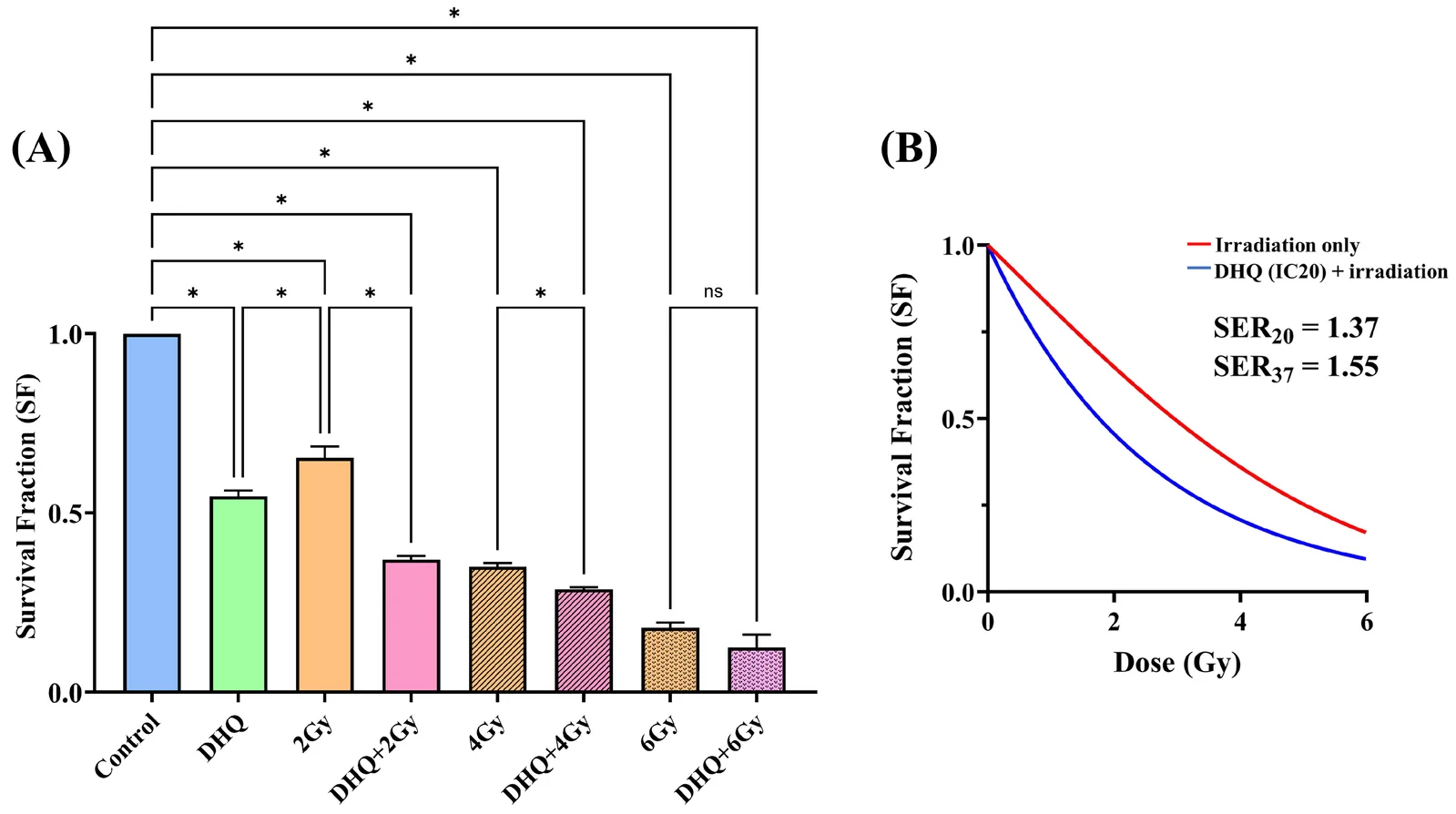
Radiosensitizing effect of dihydroquinine (DHQ) in HeLa cells. (**A**) Clonogenic survival following treatment with DHQ (IC20) and X-ray irradiation (2–6 Gy). DHQ pretreatment significantly reduced the survival fraction compared with irradiation alone at 2 and 4 Gy, whereas the reduction at 6 Gy did not reach statistical significance. (**B**) Survival curves fitted using the linear–quadratic (LQ) model, demonstrating enhanced radiosensitivity in the presence of DHQ. Sensitizer enhancement ratios (SERs), calculated at surviving fractions of 0.20 (SER20) and 0.37 (SER37), were 1.37 and 1.55, respectively. Data are presented as mean ± SD (*n* = 3). Statistical significance for selected pairwise comparisons was determined using one-way ANOVA followed by Tukey’s multiple-comparisons test. * *p* < 0.05; ns, not significant.

The radiosensitizing effect of DHQ on HeLa cells was evaluated using a clonogenic survival assay. As shown in Figure 2A, irradiation alone resulted in a dose-dependent decrease in SF, with SF values declining from 0.65 at 2 Gy to 0.18 at 6 Gy. Treatment with DHQ alone at the IC20 concentration reduced the SF to 0.55. This reduction is consistent with the higher sensitivity of the clonogenic assay compared with short-term metabolic assays, as clonogenic survival reflects long-term reproductive capacity rather than immediate cell viability. Pretreatment with DHQ before irradiation further decreased clonogenic survival, with SF values of 0.37, 0.29, and 0.13 at 2, 4, and 6 Gy, respectively. Pairwise statistical analysis showed a significant difference between DHQ alone and 2 Gy irradiation (*p* < 0.05). Importantly, DHQ pretreatment significantly reduced clonogenic survival compared with irradiation alone at 2 and 4 Gy (*p* < 0.05), whereas the reduction at 6 Gy did not reach statistical significance (*p* = 0.0678).

Survival curves were fitted using the LQ model (Figure 2B), and SER values were determined at specific survival levels. The SER20 and SER37 values were 1.37 and 1.55, respectively, indicating increased radiation sensitivity in the presence of DHQ. Taken together, these findings indicate that DHQ pretreatment reduces clonogenic survival and modifies the radiation response of HeLa cells, although the overall radiosensitizing effect is modest.

### 3.3. DHQ Increases Intracellular ROS Levels Following Irradiation

**Figure 3:**
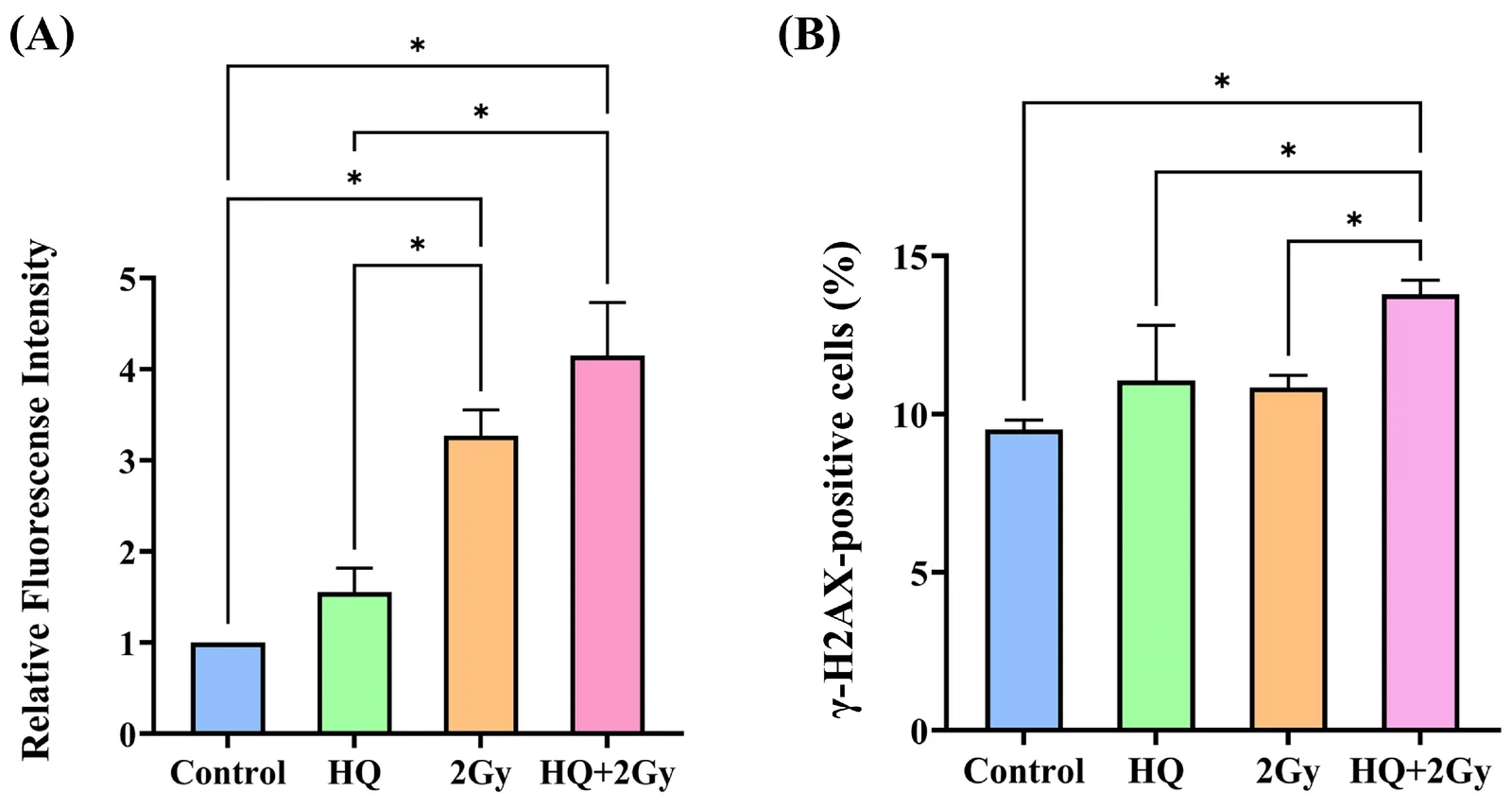
Effects of dihydroquinine (DHQ) on intracellular ROS production and γ-H2AX signaling in HeLa cells. (**A**) Intracellular ROS levels measured by DCFH-DA fluorescence following DHQ treatment and 2 Gy X-ray irradiation. DHQ pretreatment significantly increased ROS levels compared with irradiation alone. (**B**) Percentage of γ-H2AX-positive cells assessed using a semi-quantitative immunofluorescence assay. DHQ pretreatment significantly increased γ-H2AX positivity following irradiation. Data are presented as mean ± SD (*n* = 3). * *p* < 0.05.

Intracellular ROS production was measured, as shown in Figure 3A. DHQ treatment alone resulted in a slight increase in ROS levels compared with untreated controls (1.56 ± 0.26 vs. 1.00 ± 0.00). Exposure to irradiation alone significantly raised ROS levels to 3.27 ± 0.28. Importantly, cells pretreated with DHQ before irradiation exhibited the highest ROS levels (4.15 ± 0.58), significantly higher than those in both the irradiation-only and DHQ-only groups (*p* < 0.05). These findings indicate that ROS levels increased following irradiation and further increased in the presence of DHQ. These data suggest that DHQ may contribute to increased intracellular ROS generation under irradiation.

### 3.4. DHQ Enhances Radiation-Induced DNA Damage

DNA damage signaling was evaluated by γ-H2AX immunofluorescence staining after irradiation. As shown in Figure 3B, untreated control cells exhibited a baseline γ-H2AX positivity of 9.5 ± 0.3%, whereas DHQ treatment alone slightly increased this value to 11.1 ± 1.7%. Exposure to 2 Gy X-ray irradiation resulted in 10.8 ± 0.4% γ-H2AX-positive cells. Pretreatment with DHQ before irradiation produced the highest γ-H2AX positivity (13.8 ± 0.5%), which was significantly greater than that observed in the irradiation-only group (*p* < 0.05). These findings indicate that DHQ pretreatment was associated with enhanced radiation-induced γ-H2AX signaling. Because DNA damage was assessed using the percentage of γ-H2AX-positive cells rather than γ-H2AX foci enumeration, the results should be interpreted as a semi-quantitative assessment of DNA damage signaling rather than a direct quantitative measurement of DNA double-strand break formation.

### 3.5. DHQ Promotes Radiation-Induced Apoptosis

**Figure 4:**
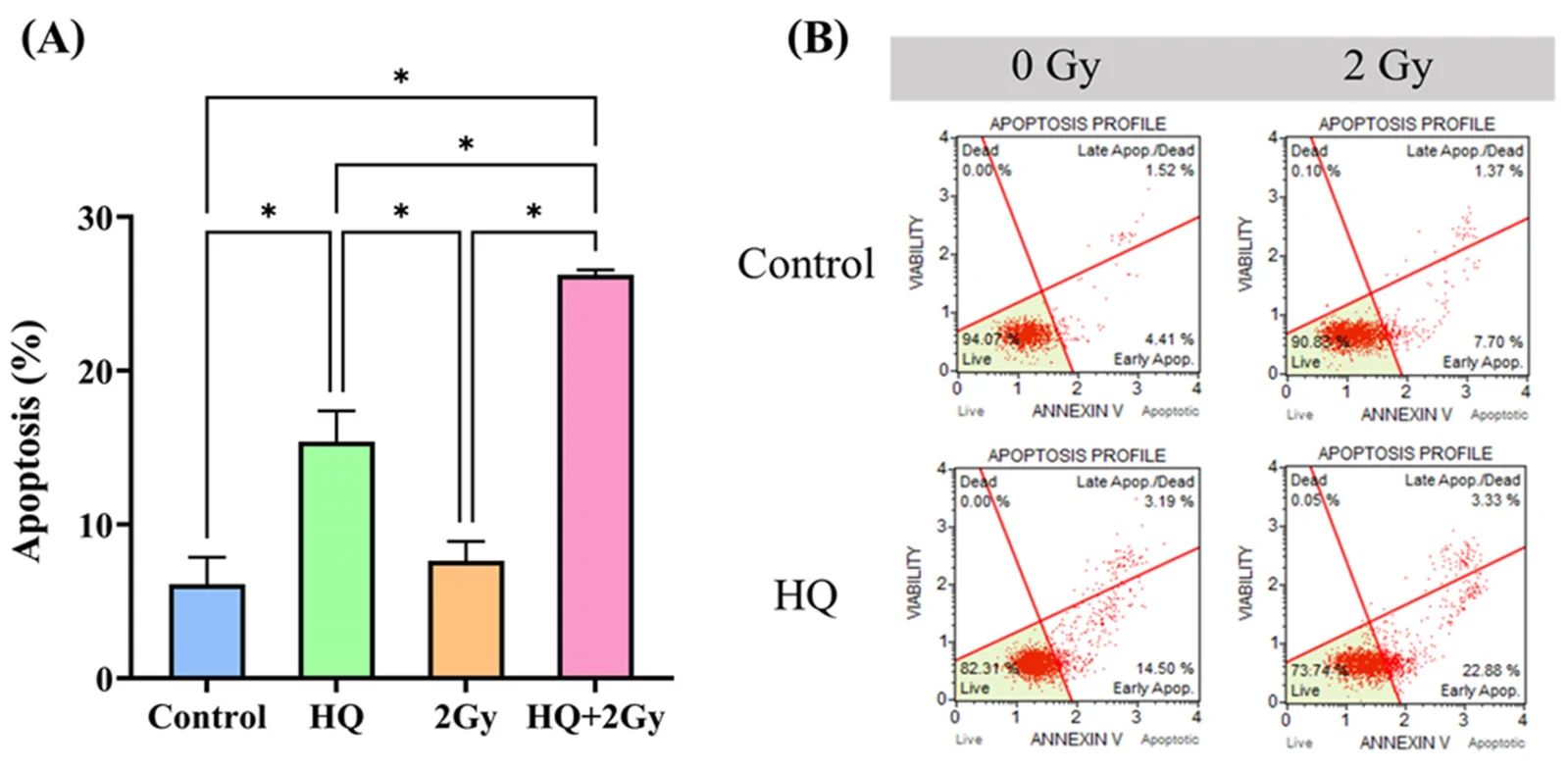
Effect of dihydroquinine (DHQ) on radiation-induced apoptosis in HeLa cells. (**A**) Quantitative analysis of apoptosis 24 h after treatment, determined using the Muse^®^ Annexin V and Dead Cell Assay. Pretreatment with DHQ followed by 2 Gy irradiation significantly increased the percentage of apoptotic cells compared with irradiation alone. (**B**) Representative Annexin V dot plots showing the distribution of viable, early apoptotic, late apoptotic, and dead cells in each group. Red dots represent individual cell events, while the gated regions indicate viable, early apoptotic, late apoptotic, and dead cell populations. Data are presented as mean ± SD (*n* = 3). * *p* < 0.05.

Apoptosis was quantitatively assessed 24 h after irradiation using the Muse^®^ Annexin V & Dead Cell Assay Kit and analyzed on the Muse^®^ Cell Analyzer. As shown in Figure 4A, untreated control cells had a low baseline apoptosis level of 6.1 ± 1.8%. DHQ alone increased apoptosis to 15.4 ± 1.9%, while irradiation alone (2 Gy) caused 7.6 ± 1.3% apoptotic cells. In particular, pretreatment with DHQ combined with irradiation resulted in the highest apoptosis rate, 26.2 ± 0.3%, which was significantly higher than those of the irradiation-only and DHQ-only groups (*p* < 0.05). These findings suggest that the combined treatment enhances apoptosis, indicating that DHQ may promote increased apoptosis under irradiation.

## 4. Discussion

In this study, we investigated the effects of DHQ, a natural Cinchona alkaloid, on the radiation response of HeLa cervical cancer cells. Using a combination of clonogenic survival analysis and mechanistic assays, our findings demonstrate that DHQ at an IC20 is associated with a modest increase in radiation response, as evidenced by reduced clonogenic survival and SER values of 1.37 and 1.55 at SF = 0.20 and 0.37, respectively. In radiobiological studies, SER values greater than 1.2 are generally considered indicative of radiosensitization [24], whereas values approaching or exceeding 1.5 suggest a moderate enhancement of the radiation response. Therefore, the SER values observed in the present study indicate that DHQ exerts a modest-to-moderate radiosensitizing effect in HeLa cells. The clonogenic survival assay is considered the gold standard for measuring radiosensitivity [25,26], and it showed that DHQ pretreatment reduced survival fractions across all radiation doses tested. The reduction was statistically significant at 2 and 4 Gy, whereas the difference at 6 Gy did not reach statistical significance. Specifically, the surviving fraction at 2 Gy decreased from 0.65 in HeLa cells exposed to radiation alone to 0.37 with DHQ pretreatment (Figure 2), indicating enhanced radiation-induced cell death.

In particular, DHQ alone decreased clonogenic survival even at an IC20 concentration based on the 24-h MTT assay. This apparent discrepancy likely reflects differences in the biological endpoints measured, as the MTT assay reflects short-term metabolic activity, while the clonogenic assay measures long-term reproductive viability. The radiosensitizing effect was assessed using dose-dependent clonogenic survival curves at 2, 4, and 6 Gy, along with the calculated SER values, rather than at a single radiation dose. The SER20 and SER37 values of 1.37 and 1.55, respectively, indicate a shift in radiation response in the presence of DHQ. The present study was not designed to determine whether the interaction between DHQ and irradiation was additive or synergistic; therefore, no such interaction is inferred from these data. However, considering the substantial effect of DHQ alone on clonogenic survival, the radiosensitizing effect should be interpreted with caution. To our knowledge, this is among the first studies to investigate the potential role of DHQ in modulating radiation responses in cervical cancer cells.

To investigate possible underlying mechanisms, we measured intracellular ROS levels. Radiation is a known factor that induces ROS generation, leading to indirect DNA damage via oxidative stress [27,28]. In our experiments, DHQ alone caused a minor increase in ROS levels, whereas irradiation significantly increased them. In particular, combining DHQ with irradiation yielded the highest ROS signals. These data suggest that DHQ may disrupt redox homeostasis and amplify oxidative stress during irradiation, consistent with previous findings that reactive oxygen species are key factors in tumor radiosensitization [29]. However, because ROS was only measured at a single time point, our understanding of its dynamic regulation remains limited. These data align with previous studies indicating that assessments taken at a single time point may not fully reflect the complex balance between ROS production and scavenging [30]. However, these findings suggest that DHQ may lower the cellular threshold for radiation-induced oxidative damage.

In accordance with elevated oxidative stress, we observed higher γ-H2AX levels in positive cells in the treatment group receiving both treatments (Figure 3B). γ-H2AX is a common marker of DNA double-strand breaks (DSBs) [31], which are among the most lethal forms of radiation-induced DNA damage. While irradiation alone increased the number of γ-H2AX–positive cells, pretreatment with DHQ further increased the γ-H2AX signal, suggesting that DHQ may enhance DNA damage signaling during irradiation. However, in this study, DNA damage was evaluated by the percentage of γ-H2AX–positive cells rather than by counting individual foci. Although foci counting is generally considered a more precise method for quantifying DNA double-strand breaks, assessing the proportion of positive cells offers a practical, widely used alternative, particularly when fluorescence signals are strong or overlap [32,33,34]. γ-H2AX, a phosphorylated form of histone H2AX, is rapidly induced in response to DSBs and serves as a sensitive biomarker of DNA damage, with signal amplification facilitating detection by fluorescence microscopy [33,34,35]. Consequently, using a percentage-based method can still reliably indicate overall DNA damage under specific experimental conditions.

Nevertheless, conventional fluorescence microscopy has inherent limitations, including difficulty in resolving closely spaced or overlapping foci, which may affect accurate quantification. In addition, manual analysis may introduce observer-related variability [32]. Therefore, these results should be considered a semi-quantitative evaluation of DNA damage rather than a precise measurement. Additionally, γ-H2AX was measured at only a single time point after irradiation, which does not reflect the full temporal pattern of DNA damage formation and repair. Previous research has shown that the kinetics of γ-H2AX expression and DNA repair over time are important for accurately assessing radiation-induced biological effects and cellular damage [36].

Consistent with the observed increase in DNA damage, the combined treatment resulted in a higher apoptotic rate than either DHQ or irradiation alone (Figure 4). Although DHQ alone induced measurable apoptosis, the combined treatment resulted in a higher apoptotic rate than either treatment alone. However, the apoptosis assay was not designed to distinguish additives from synergistic interactions. Therefore, the increased apoptosis observed following combined treatment should be interpreted as an enhanced apoptotic response rather than evidence of synergism. Mechanistically, this effect may be associated with increased oxidative stress and DNA damage, as elevated ROS levels can amplify radiation-induced cellular damage and activate apoptotic signaling pathways [37]. Additionally, the increased γ-H2AX positivity (Figure 3B) suggests that DHQ may increase persistent DNA damage or delay DNA repair following irradiation, since prolonged DNA double-strand breaks can trigger apoptosis [38]. However, the observed increases in ROS, γ-H2AX positivity, and apoptosis represent associated cellular responses and do not establish that DHQ and ionizing radiation act through identical or competing molecular mechanisms. Further mechanistic studies are required to determine how DHQ modulates radiation-induced cellular responses.

Taken together, the findings of this study are consistent with a role for DHQ in modulating cellular responses to ionizing radiation by altering multiple biological processes, including increased oxidative stress, heightened DNA damage signaling, and elevated apoptosis. Rather than acting as a potent radiosensitizer, DHQ appears to have a moderate effect on radiation response. In clinical practice, platinum-based compounds are well-established radiosensitizers for cervical cancer. Cisplatin combined with radiotherapy remains the standard approach for treating locally advanced cervical cancer, whereas carboplatin has been explored as an alternative, particularly for patients unable to tolerate cisplatin. In this context, DHQ should be considered an exploratory radiosensitizing candidate rather than a replacement for established platinum-based chemoradiotherapy. Further studies are required to determine its potential therapeutic relevance [39,40]. From a translational perspective, natural compounds have gained increasing attention as radiosensitizing agents due to their diverse biological activities and favorable safety profiles [15]. Although the magnitude of radiosensitization observed in this study is modest, the consistency of effects across multiple biological endpoints suggests that DHQ may serve as a promising adjunct in combination strategies targeting oxidative stress and DNA damage response pathways.

The radiosensitizing effects observed for DHQ are consistent with previous reports describing naturally derived radiosensitizers. Curcumin has been shown to enhance radiation-induced cytotoxicity by increasing ROS production, amplifying DNA damage, and inducing apoptosis [41,42]. Similarly, resveratrol has been reported to potentiate radiation responses by modulating oxidative stress and apoptotic signaling pathways [43,44]. Quercetin has also demonstrated radiosensitizing properties, including increased oxidative stress, accumulation of DNA damage, and enhanced apoptosis following irradiation [45]. Similar to these phytochemicals, DHQ was associated with increased intracellular ROS levels, elevated γ-H2AX positivity, and enhanced apoptosis following irradiation. Although direct comparisons among studies should be interpreted with caution due to differences in experimental models and treatment conditions, these findings support the growing interest in naturally derived compounds as adjunctive agents for radiotherapy [15].

Overall, this study shows that DHQ slightly improves the radiation response of cervical cancer cells when exposed to clinically relevant megavoltage X-ray irradiation. The findings of decreased clonogenic survival, along with elevated ROS production, γ-H2AX positivity, and apoptosis, justify further research into DHQ as a natural radiosensitizer. Nevertheless, several limitations should be acknowledged. First, all experiments were performed in a single cervical cancer cell line (HeLa). Second, although preliminary cytotoxicity was evaluated in human fibroblasts (Supplementary Figure S1), the effects of DHQ combined with irradiation were not investigated in normal cells. Therefore, whether DHQ selectively enhances radiation responses in cancer cells while sparing normal tissues remains unknown and warrants further investigation. Third, the present study evaluated ROS generation, γ-H2AX positivity, and apoptosis as cellular endpoints but did not investigate the molecular pathways underlying these responses. Therefore, whether DHQ and irradiation act through shared, distinct, or interacting molecular mechanisms remains to be determined. Finally, this in vitro study did not evaluate γ-H2AX foci, DNA repair kinetics, or in vivo therapeutic responses. Future studies should validate the effects of DHQ in additional cancer models and preclinical systems, evaluate lower DHQ concentrations to optimize the balance between intrinsic cytotoxicity and radiation-response enhancement, and investigate the molecular mechanisms underlying the interaction between DHQ and ionizing radiation.

## 5. Conclusions

In summary, this study provides preliminary evidence that DHQ, at the IC20 concentration, modestly modifies the radiation response of HeLa cervical cancer cells. Pretreatment with DHQ reduced clonogenic survival and yielded SER values of 1.37 and 1.55 at SF of 0.20 and 0.37, respectively, indicating a shift in the radiation survival response in the presence of DHQ. Combined DHQ and irradiation treatment was associated with increased intracellular ROS levels, γ-H2AX positivity, and apoptosis. These findings indicate that DHQ affects cellular responses to ionizing radiation across multiple biological endpoints. However, considering the substantial effect of DHQ alone on clonogenic survival, the observed radiation-response modification should be interpreted cautiously. Further studies using optimized DHQ concentrations, additional experimental models, and more detailed mechanistic analyses are required to establish the potential of DHQ as a radiosensitizing agent.

## Data Availability

The original contributions presented in this study are included in the article. Further inquiries can be directed to the corresponding author.

## Supplementary Materials

The following supporting information can be downloaded at https://www.mdpi.com/article/10.3390/cells15161484/s1. Figure S1. Cytotoxicity of dihydroquinine (DHQ) in human fibroblasts after 24 h. Human fibroblasts were exposed to DHQ (10–50 µg/mL) for 24 h, and cellular metabolic activity was determined using the MTT assay. Untreated cells and DMSO vehicle controls (0.75% and 1.5%) were included. Data are presented as mean ± SD (*n* = 3). * *p* < 0.05.
