# Supplementary material for: Dihydroquinine Enhances Radiosensitivity in Cervical Cancer Cells Accompanied by Radiation-Induced Cellular Responses
Source: Cells. 2026 Aug 18;15(16):1484. doi: 10.3390/cells15161484 (PMC13511470; doi:10.3390/cells15161484)
Supplement: Supplementary file 1 [file cells-15-01484-s001.zip › cells-4493864-supplementary.pdf]

Supplementary materials

Dihydroquinine Enhances Radiosensitivity in Cervical Cancer Cells Accompanied by Radiation-Induced Cellular Responses

Ausanai Prapan, Pimvaree Aissara, Peerawit Soonthornchookiat, Chanyatip Suwannasing, Jirapas Jongjitwimol, Chatrawut Pattaweerakul, Yu Xiong, Saranya Chaiwaree, and Hans Bäumler*

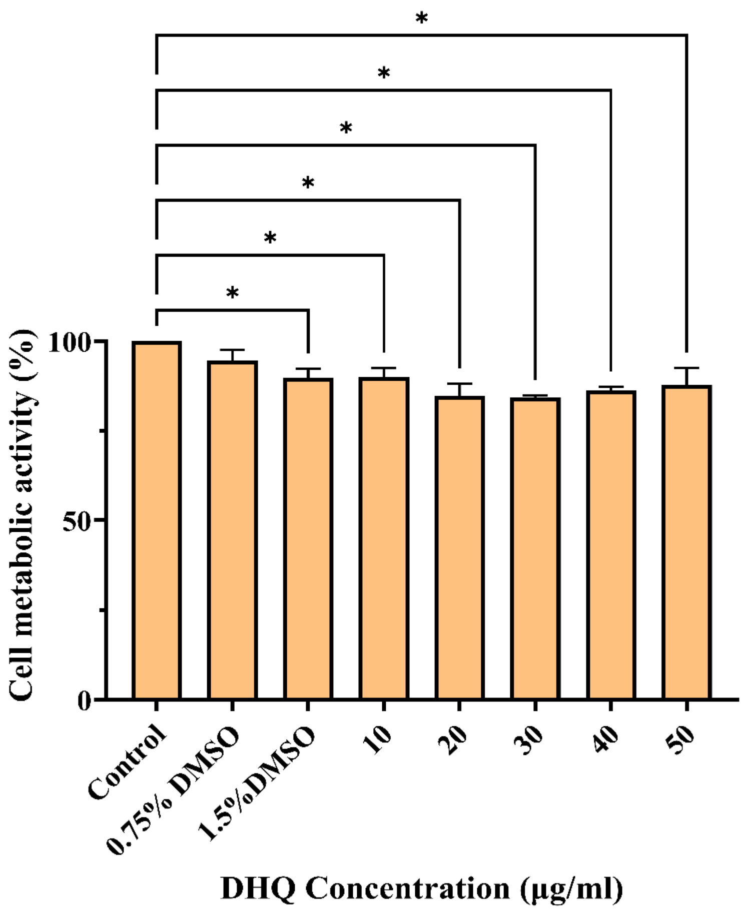

Figure S1. Cytotoxicity of dihydroquinine (DHQ) in human fibroblasts after 24 h. Human fibroblasts were exposed to DHQ (10–50 μg/mL) for 24 h, and cellular metabolic activity was determined using the MTT assay. Untreated cells and DMSO vehicle controls (0.75% and 1.5%) were included.

Data are presented as mean ± SD (n = 3). * $p < 0.05$.
